# Differences in characteristics between people with tinnitus that seek help and that do not

**DOI:** 10.1038/s41598-021-01632-5

**Published:** 2021-11-25

**Authors:** M. M. Rademaker, I. Stegeman, A. E. M. Brabers, J. D. de Jong, R. J. Stokroos, A. L. Smit

**Affiliations:** 1 Department of Otorhinolaryngology and Head and Neck Surgery, University Medical Center Utrecht, Utrecht, The Netherlands; 2 UMC Utrecht Brain Center, Utrecht University, Utrecht, The Netherlands; 3 Department of Ophthalmology, University Medical Center Utrecht, Utrecht, The Netherlands; 4 Epidemiology and Data Science, Amsterdam University Medical Centers, University of Amsterdam, Amsterdam, The Netherlands; 5 Nivel (Netherlands Institute for Health Services Research), Utrecht, The Netherlands; 6 Care and Public Health Research Institute, Maastricht University, Maastricht, The Netherlands

**Keywords:** Epidemiology, Neurology

## Abstract

Knowledge on characteristics of people that seek help for tinnitus is scarce. The primary objective of this study was to describe differences in characteristics between people with tinnitus that seek help compared to those who do not seek help. Next, we described differences in characteristics between those with and without tinnitus. In this cross-sectional study, we sent a questionnaire on characteristics in different domains; demographic, tinnitus-specific, general- and psychological health, auditory and noise- and substance behaviour. We assessed if participants had sought help or planned to seek help for tinnitus. Tinnitus distress was defined with the Tinnitus Functional Index. Differences between groups (help seeking: yes/no, tinnitus: yes/no) were described. 932 people took part in our survey. Two hundred and sixteen participants were defined as having tinnitus (23.2%). Seventy-three of those sought or planned to seek help. A constant tinnitus pattern, a varying tinnitus loudness, and hearing loss, were described more frequently in help seekers. Help seekers reported higher TFI scores. Differences between help seekers and people not seeking help were mainly identified in tinnitus- and audiological characteristics. These outcomes might function as a foundation to explore the heterogeneity in tinnitus patients.

## Introduction

Although the word tinnitus originates from the Latin word ‘tinnire’, which translates into ‘’to ring’’, people with tinnitus can experience many different sounds such as buzzing or humming^1^. Some people even describe to hear the sound of a complete orchestra playing in their ear^2^. Not only is there variance in the nature of the sound, also the location, pitch and loudness differ between patients. Besides, the consequences of tinnitus on daily life vary widely among individuals due to its associated co-morbidities such as concentration-, sleep- or mental health problems^3^. In a recent paper the authors therefore advocate to differentiate between the experience of tinnitus, and the associated suffering due to the tinnitus, which they refer to as tinnitus disorder^4^. All these factors contribute to the complexity and heterogeneity of tinnitus^3^. Tinnitus prevalence numbers range between 5.1 and 42.7% due to differences in definitions and the studied populations^5^.

It is commonly believed that one of the explanations of the heterogeneity might be the existence of subtypes of tinnitus patients. Several attempts have been made to define these subtypes, but clinically usable types remain to be found^6^. In a recent review on tinnitus subtyping, the authors identified 64 articles that had reported on tinnitus subtyping^6^. They extracted 94 different variables which were processed in a framework of the most commonly used variables in subtyping. Tinnitus severity, hearing ability, age, and depressive symptoms were found to be the top four variables that were significant or important for classification^6^. However such characteristics can cover many domains such as demographic, audiological or psychological measures. In order to understand the role of these characteristics in tinnitus patients, we first need to know the differences between people with and without tinnitus. The development of the ESIT-SQ emphasizes this. One of their objectives was to: “create a questionnaire that would allow standardized data collection from the entire adult population, tinnitus and non-tinnitus, which are essential for investigating mechanisms associated with tinnitus^7^, p. 3.

Another challenge of the heterogeneous aspect of tinnitus is that there is a great variation in the help seeking behavior of those affected. Understanding the differences between those that seek help versus those that do not seek help for their tinnitus might help to illuminate the heterogeneity issue. What are the reasons from a transition from experiencing tinnitus into having tinnitus disorder^4^? A Swedish survey study, performed in 2000 in a randomly selected population sample, analyzed characteristics related to seeking help. They showed that help seeking tinnitus participants had higher scores in questionnaires assessing psychological problems such as anxiety and negative mood compared to non-help seeking tinnitus patients^8^. An Israeli study, from 1993 in young male active army personnel (n=100), with both patients with and without tinnitus, identified differences between those that sought help and those that did not. Help seekers had poorer coping techniques, and their psychiatric symptomatology was more severe than the people that did not seek help^9^. A third study in tinnitus patients from a hospital setting in Sweden performed in 1993 identified differences between so-called “complainers” and “non-complainers”. “Complainers” more often reported a combination of tinnitus sounds and had more problems with concentration than ‘’non-complainers’’^10^.

Combining the knowledge about the differences in characteristics between those with and without tinnitus, and those seeking help versus those not seeking help for their tinnitus is of importance. We believe descriptive studies of differences in both study groups will help the international tinnitus community in their search for tinnitus subtypes and in the ultimate goal to create effective treatments for specific subgroups affected. Besides this, this knowledge is of importance to optimize health care in terms of counselling and diagnostics of those affected.

Therefore, in this study our main objective was to describe the differences in characteristics between people with tinnitus that seek help versus those who do not seek help in a random sample of the Dutch general population. Next, to be able to interpret outcomes as a secondary aim we compared characteristics of people with and without tinnitus. Differences in demographic-, tinnitus-specific-, general health-, psychological health-, audiological characteristics, and characteristics about noise- and substance behaviour were assessed.

## Methods

This paper was written according to the Strengthening the Reporting of Observational Studies in Epidemiology (STROBE) statement^11^. (Supplementary Methods S1).

### Study design and population

For this observational study we prospectively gathered data by means of a questionnaire send to Dutch adults. They were members of the Dutch Health Care Consumer panel^12^.

“The aim of the Nivel Dutch Health Care Consumer Panel (DHCCP) is to measure, at national level, opinions on and knowledge about health care and the expectations and experiences with health care. The Consumer Panel is a so-called ‘access panel’. An access panel consists of a large number of persons who have agreed to answer questions on a regular basis. In addition, many background characteristics of these persons (for example age, level of education, income, self-reported general health) are known^12^”. At the time of this study (January 2020), the panel consisted of approximately 12,000 people aged 18 years and older. “From the access panel samples can be drawn for every separate survey. It is not possible for people to sign up on their own initiative. The panel is renewed on regular basis. Renewal is necessary to make sure that members do not develop specific knowledge of, and attention for, healthcare issues, and that no ‘questionnaire fatigue’ occurs. Moreover, renewal compensates for panel members who, for example, have died or moved without informing the panel about the new address^12^”.

This study is part of a larger study on tinnitus prevalence, characteristics and health care usage. The study sample therefore entails those DHCCP panel members (N = 2251), who agreed to combine their survey answers with health care consumption data as registered by their general practitioner^13^**.** A previously published study on tinnitus prevalence was based on the same data^14^**.**

### Outcome assessment

#### Logistics

A questionnaire was sent to panel members of the Dutch Health Care Consumer Panel. The questionnaire was sent via postal services or online. This depended on the preference of the panel member. The postal survey was sent on 14-01-2020. One postal reminder was sent on 30-01-2020. The online survey was sent on the 16-01-2020. Two online reminders were sent on 23-01-2020 and 30-01-2020. The survey was closed on 14-02-2020. Please find the questionnaire in Supplementary Methods S2.

#### Questionnaire outline

The survey was created by MR (medical doctor), AS (ENT surgeon), IS (epidemiologist) and AB (senior researcher Nivel). Characteristics among different domains were collected: demographic, tinnitus-specific, general health, psychological health, audiological, and noise- and substance behaviour. The full questionnaire can be found in Supplementary Methods S2. The overall survey structure was based on the European School for Interdisciplinary Tinnitus Research Screening Questionnaire (ESIT-SQ), which consists of two parts^7^. Part one consist of 17 questions regarding individual characteristics in people with or without tinnitus. The second part is only meant for people with tinnitus. It consists of 22 questions regarding tinnitus characteristics. The set-up of our survey was similar: a part to be answered by all participants, and a part that was specifically for those that had tinnitus. For the part to be answered by all participants, we directly used or used a variation on 13 of 17 questions of the ESIT-SQ part A. Survey items about the characteristics of tinnitus were based on the Tinnitus Sample Case History Questionnaire (TSCHQ) and the ESIT-SQ part B^7,15,16^. The TSCHQ consists of 35 questions concerning tinnitus history and tinnitus characteristics. We did not use the full versions of one of both questionnaires due to space limitations. Questions were either an exact copy of one of two questionnaires or questions/answer options were combined. Twelve questions were based or an exact copy of the 22 questions of ESIT-SQ part B. Twenty-three questions were based on or an exact copy of the 35 questions of the TSCHQ.

#### Demographics

Demographic data were gathered when people became a member of the panel and were provided by Nivel for this study. These include data about educational level, marital status, social position (e.g. employed/unemployed/student), ethnicity, age (calculated at date of sending of the questionnaire), gender, and net income of the households of the participating panel members, self-reported general health and self-reported mental health.

#### Tinnitus classification and definitions

We assessed the presence of tinnitus with three questions. We described tinnitus as *Tinnitus is the hearing of e.g. a beep, whistle, hissing, zoom or another sound without the actual presence of the sound in your surroundings. This can last for a very short amount of time or a whole day.* First, the participants were asked whether they experienced tinnitus over the last year. Next, a question about duration was asked (tinnitus lasting < 5 min, 5–60 min, ≥ 60 min or continuously). The third question was about the frequency of the experienced sound (daily or almost daily, weekly, monthly, less than once a year). We subsequently defined people as having tinnitus when they experienced the sound for 5–60 min (daily or almost daily or weekly), or tinnitus for ≥ 60 min or continuously (daily or almost daily or weekly or monthly). This was based on literature and expert opinion^5^.

#### Tinnitus characteristics

The following items were assessed: whether the participant had sought help for tinnitus or planned to seek help and the source of the help, tinnitus pattern, subjective problem of tinnitus, acute or chronic tinnitus (< 3 months, 3–6 months, ≥ 6 months), manner of the tinnitus start, number of different sounds, pulsatile nature, whether the tinnitus varied in loudness, the pitch and location of the tinnitus, the intrusiveness of the tinnitus, influencing factors, potential causes.

#### Definition of help seeking tinnitus participants

We defined participants as help seeking tinnitus participants, if they had sought help in the past or planned to seek help for their tinnitus.

#### Tinnitus distress

The impact of tinnitus on daily life was assessed with the multi-item Dutch translation of the Tinnitus Functional Index (TFI) questionnaire^17,18^. This questionnaire consists of 25 questions, with answers on an 11-point Likert scale. The final score ranges between 0 and 100; a score between 0 and 17 can be interpreted as not a problem, 18–31 as a small problem, 32–53 as a moderate problem, 54–72 as a big problem and 73–100 as a very big problem. The 25 questions of the TFI are a combination of scores of impact on daily life out of eight subcategories, intrusiveness, sense of control, cognition, sleep, hearing, relaxation, quality of life and emotions, each covered by 3–4 questions. The TFI was developed and validated in the United States of America and translated and validated from English to Dutch in 2014. The Dutch translation by Tromp et al. holds a high internal consistency (Cronbach’s alpha of 0.91)^18^.

#### General health characteristics

The following items were asked in the questionnaire to assess general health: the presence of chronic pain, family history of certain diseases, and presence of certain diseases as diagnosed by a doctor.

#### Psychological health characteristics

Symptoms of anxiety and depression were measured with the Hospital Anxiety and Depression Scale (HADS), The HADS is a 14-item questionnaire that measures symptoms of anxiety (HADS-A; seven items) and depression (HADS-D; seven items) on a four point scale^20^. The HADS was translated and validated to Dutch by Spinhoven et al (Cronbach’s alpha ranges between 0.71 and 0.90 for both subscales and the total scale)^19^. The total scores range from 0 to 21. A score of 8 or higher indicates a potential anxiety or depression^19,20^.

#### Audiological characteristics

The following items were assessed in the questionnaire to assess audiological characteristics: hyperacusis, presence of hearing problems, use of hearing aids/cochlear implants/sound generator or tinnitus maskers, and auditory hallucinations.

#### Characteristics on noise- and substance behavior

The following items were assessed in the questionnaire to assess noise and substance behaviour: the use of head- or earphones, exposure to potential damaging sound levels (subjectively judged), the use of hearing protection, smoking habits, drug use and alcohol consumption.

#### Data handling and ethics

Data are analyzed anonymously and the privacy of the panel members is guaranteed, as is described in the privacy policy of the Dutch Health Care Consumer Panel. This complies with the General Data Protection Regulation (GDPR). According to Dutch legislation, neither obtaining informed consent nor approval by a medical ethics committee is obligatory for conducting research through the panel (CCMO, 2020)^12^. The Medical Research Ethics Committee (MREC) of the University Medical Center Utrecht (UMC Utrecht) confirmed on November 20th 2019, that the Medical Research Involving Human Subjects Act (WMO) does not apply to this study and that therefore official approval by the MREC is not required under the Human Subjects Act (MREC local protocol number 19-745). This study was performed according to the declaration of Helsinki.

#### Statistical analysis

Statistical analyses were performed with SPSS version 26.0.0.1^21^. Normality of variables was visually assessed. Frequencies, means, standard deviation (SD), medians and interquartile ranges (IQR) were calculated for the total study group, participants with or without tinnitus and help seeking versus non-help seeking participants. A *p* value of 0.05 or lower was considered statistically significant. Logistic regression was only performed for a subset of the characteristics. These were based on known risk factors from the literature for tinnitus and expert opinion. The following characteristics were assessed, these were based on the answers to the different questions in the survey: tinnitus pattern, subjective problem of tinnitus, duration of tinnitus, varying loudness, tinnitus intrusiveness, TFI score and TFI grades, chronic pain, HADS-A, HADS-D, hyperacusis, hearing problems, the use of different hearing aids, auditory hallucinations, use of head/ear phones, potential damaging sound levels, use of hearing protection, gender, age and educational level.

## Results

### Study sample

Of the 2251 panel members who were invited to participate in the survey 932 (41.4%) filled out the questionnaire. The median age of the participants was 67.0 (IQR 17) years and 52.4% was female (Table 1).

**Table 1:** Demographic characteristics.

Demographic	Total %	Tinnitus %		OR (95% CI)	Help %		OR (95% CI)
		No	Yes		No	Yes	
**Gender**							
Male	444 (47.6)	309 (44.8)	124 (57.4)	Ref	76 (53.5)	47 (64.4)	Ref
Female	488 (52.4)	381 (55.2)	92 (42.6)	0.60 (0.44–0.82)*	66 (46.5)	26 (35.6)	0.64 (0.36–1.14)
Missing	0 (0.0)	0 (0.0)	0 (0.0)		0 (0.0)	0 (0.0)	
**Age**^a^							
	67.0 (17)	67 (19)	66.5 (15)	1.01 (0.996–1.021)	66 (16)	69 (13)	1.02 (0.997–1.052)
N =	932	690	216		142	73	
**Age categorized**							
18–39	29 (3.1)	28 (4.1)	1 (0.5)		1 (0.7)	0 (0.0)	
40–64	349 (37.4)	261 (37.8)	85 (39.4)		60 (42.3)	25 (34.2)	
65 +	554 (59.4)	401 (58.1)	130 (60.2)		81 (57.0)	48 (65.8)	
Missing	0 (0.0)	0 (0.0)	0 (0.0)		0 (0.0)	0 (0.0)	
**Highest completed educational level**							
Low	157 (16.8)	118 (17.1)	27 (12.5)	Ref	16 (11.3)	11 (15.1)_	Ref
Middle	337 (40.5)	290 (42.0)	78 (36.1)	1.18 (0.72–1.91)	56 (39.4)	21 (28.8)	0.55 (0.22–1.37)
High	372 (39.9)	264 (38.3)	104 (48.1)	1.72 (1.07–2.77)*	66 (46.5)	38 (52.1)	0.84 (0.25–1.99)
Missing	26 (2.8)	18 (2.6)	7 (3.2)		4 (2.8)	3 (4.1)	
**Marital status**							
Married	617 (66.2)	449 (65.1)	152 (70.4)		103 (72.5)	49 (67.1)	
Divorced	73 (7.8)	48 (7.0)	23 (10.6)		11 (7.7)	12 (16.4)	
Widowed	90 (9.7)	70 (10.1)	14 (6.5)		11 (7.7)	3 (4.1)	
Never been married	146 (15.7)	120 (17.4)	24 (11.1)		15 (10.6)	9 (12.3)	
Missing	6 (0.6)	3 (0.4)	3 (1.4)		2 (1.4)	0 (0.0)	
**Social position**							
School/studying	17 (1.8)	13 (1.9)	4 (1.9)		4 (2.8)	0 (0.0)	
Employed	410 (44.0)	305 (44.2)	98 (45.4)		70 (49.3)	28 (38.4)	
Unemployed (work seeking)	31 (3.3)	22 (3.2)	9 (4.2)		5 (3.5)	4 (5.5)	
Incapacitated	36 (3.9)	26 (3.8)	9 (4.2)		7 (4.9)	2 (2.7)	
Housewife/husband	118 (12.7)	90 (13.0)	20 (9.3)		16 (11.3)	4 (5.5)	
Retired	393 (42.2)	292 (42.3)	86 (39.8)		50 (35.2)	35 (47.9)	
Other	39 (4.2)	30 (4.3)	9 (4.2)		6 (4.2)	3 (4.1)	
Missing	0 (0.0)		0 (0.0)				
**Ethnicity**^b^							
Native Dutch	873 (93.7)	653 (94.6)	197 (91.2)		129 (90.8)	67 (91.8)	
Western non-native Dutch	52 (5.6)	31 (4.5)	18 (8.3)		13 (9.2)	5 (6.8)	
Non-western non-native Dutch	6 (0.6)	5 (0.7)	1 (0.5)		0 (0.0)	1 (1.4)	
Missing	1 (0.1)	1 (0.1)	0 (0.0)		0 (0.0)	0 (0.0)	
**Net income**							
€ 0–2100	320 (34.3)	233 (33.8)	67 (31.0)		41 (28.9)	25 (34.2)	
€ 2100–2300	70 (7.5)	56 (8.1)	13 (6.0)		10 (7.0)	3 (4.1)	
€ 2300–3300	263 (28.2)	191 (27.7)	69 (31.9)		39 (27.5)	30 (41.1)	
€> 3300	241 (25.9)	176 (25.5)	64 (29.6)		52 (36.6)	12 (16.4)	
Don’t want to say	0 (0.0)	0 (0.0)	0 (0.0)		0 (0.0)	0 (0.0)	
Missing	38 (4.1)	34 (4.9)	3 (1.4)		0 (0.0)	3 (4.1)	

Please see Supplementary Table S2 for answer to social position, other.

^a^Median (IQR) *p < 0.05.

^b^Based on country of birth of the parents.

### Tinnitus and its characteristics

Out of the 932 participants, 216 (23.2%, 26 missing) were classified as having tinnitus based on the set criteria of duration and frequency of the experienced sound (Table 2). Out of these 216 tinnitus participants (91.7%, 1 missing,) 198 experienced their tinnitus for 6 months or more. The total TFI-score could be calculated for 212 tinnitus participants (4 missing) and the median total score was 16.6 (IQR 21.8) (Table 3).

**Table 2:** Tinnitus participants and help seeking participants with tinnitus.

	N	%
**Tinnitus for 5–60 min**		
*Daily or almost daily*	*20*	*31.3*
*Weekly*	*20*	*31.3*
Monthly	16	25.0
≤ 1 time per year	5	7.8
Missing	3	4.7
**Tinnitus for ≥ 60 or continuously**		
*Daily or almost daily*	*153*	*80.5*
*Weekly*	*13*	*6.8*
*Monthly*	*10*	*5.3*
≤ 1 time per year	3	1.6
Missing	11	5.8
**Tinnitus participant**		
Yes	216	23.2
No	690	74.0
Missing	26	2.8
**Sought help**		
Yes	72	33.3
No	143	66.2
Missing	1	0.5
**If no. plans to seek help**		
Yes	1	0.7
No	140	97.9
Missing	2	1.4
**Source of treatment**		
Psychiatric	0	0.0
Psychologic	6	8.2
Audiological	21	28.8
Physiotherapy	2	2.7
Self-management	2	2.7
Alternative medicine	8	11.0
Doctor	39	53.4
Other	7	9.6
Missing	9	12.3
**Help seeking tinnitus participant**		
Yes	73	33.8
No	142	65.7
Missing	1	0.5

Tinnitus participant were defined as experiencing tinnitus for 5–60 min daily or almost daily, or weekly or 60 min or more or continuously daily or almost daily, weekly or monthly. (These are written cursive)

Please see Supplementary Table S2 for answer to type of tinnitus help, other.

**Table 3:** Tinnitus characteristics.

Tinnitus characteristics	Experiencing tinnitus n (%)	Tinnitus help seeking n (%)			OR (95% CI)
		No	Yes		
**Pattern**					
Constant	135 (62.5)	77 (54.2)		58 (79.5)	3.26 (1.69–6.30)*
Intermittent	80 (37.0)	65 (45.8)		15 (20.5)	Ref
Missing	1 (0.5)	0 (0.0)		0 (0.0)	
**Subjective problem of tinnitus**					
No	51 (23.6)	48 (33.8)		3 (4.1)	Ref
Small	105 (48.6)	70 (49.3)		35 (47.9)	8.0 (2.33–27.51)*
Reasonable	43 (19.9)	20 (14.1)		23 (31.5)	18.4 (4.96–68.29)
Large	12 (5.6)	4 (2.8)		8 (11.0)	32.0 (6.00–170.61)*
Very large	4 (1.9)	0 (0.0)		4 (5.5)	Error
Missing	1 (0.5)	0 (0.0)		0 (0.0)	
**Tinnitus begin**					
< 3 months	8 (3.7)	7 (4.9)		1 (1.4)	Ref
3 till 6 months	9 (4.2)	8 (5.6)		1 (1.4)	0.88 (0.05–16.74)
≥ 6 months	198 (91.7)	127 (89.4)		71 (97.3)	3.91 (0.47–32.45)
Missing	1 (0.5)	0 (0.0)		0 (0.0)	
**# of different sounds**					
1	167 (77.3)	117 (82.4)		50 (68.5)	
More than 1	48 (22.2)	25 (17.6)		23 (31.5)	
Missing	1 (0.5)	0 (0.0)		0 (0.0)	
**Pulsatile**					
Yes	23 (10.6)	15 (10.6)		8 (11.0)	
No	170 (78.7)	114 (80.3)		56 (76.7)	
Missing	23 (10.6)	13 (9.2)		9 (12.3)	
**Manner of tinnitus’ start**					
Gradually	147 (68.1)	103 (72.5)		44 (60.3)	
Suddenly	61 (28.2)	34 (23.9)		27 (37.0)	
Missing	8 (3.7)	5 (3.5)		2 (2.7)	
**Varying loudness**					
Yes	106 (49.1)	58 (40.8)		48 (65.8)	2.97 (1.62–5.46)*
No	101 (46.8)	79 (55.6)		22 (30.1)	Ref
Missing	9 (4.2)	5 (3.5)		3 (4.1)	
**Pitch**					
High	76 (35.2)	55 (38.7)		21 (28.8)	
Average	75 (34.7)	48 (33.8)		27 (37.0)	
Low	42 (19.4)	26 (18.3)		16 (21.9)	
I don’t know	16 (7.4)	9 (6.3)		7 (9.6)	
Missing	7 (3.2)	4 (2.8)		2 (2.7)	
**Intrusiveness**^a^					
	4 (5)	3 (4)		5 (4)	1.298 (1.15–1.47)*
N =	215	142		73	
**Location**					
Right ear	17 (7.9)	9 (6.3)		8 (11.0)	
Left ear	30 (13.9)	16 (11.3)		14 (19.2)	
Both > right ear^b^	28 (13.0)	18 (12.7)		10 (13.7)	
Both > left ear^c^	37 (17.1)	25 (17.6)		12 (16.4)	
Both equal	79 (36.6)	58 (40.8)		21 (28.8)	
Inside head	39 (18.1)	23 (16.2)		16 (21.9)	
Other	2 (0.9)	1 (0.7)		1 (1.4)	
Missing	9 (4.2)	5 (3.5)		3 (4.1)	
**Influence**					
Presence of loud sounds	57 (26.4)	36 (25.4)		21 (28.8)	
Music or surrounding sounds	69 (31.9)	44 (31.0)		25 (34.2)	
Head or neck movements	15 (6.9)	10 (7.0)		5 (6.8)	
Touching the head with arms/hands	5 (2.3)	2 (1.4)		3 (4.1)	
Sleep during the day	13 (6.0)	7 (4.9)		6 (8.2)	
Good sleep quality	34 (15.7)	20 (14.1)		14 (19.2)	
Stress	47 (21.8)	28 (19.7)		19 (26.0)	
Medicine	5 (2.3)	4 (2.8)		1 (1.4)	
Hearing aids	24 (11.1)	10 (7.0)		14 (19.2)	
Nothing	68 (31.5)	48 (33.8)		20 (27.4)	
Other	22 (10.2)	9 (6.3)		13 (17.8)	
Missing	2 (0.9)	1 (0.7)		0 (0.0)	
**Potential cause**					
Flu, cold or other infection	22 (10.2)	10 (7.0)		12 (16.4)	
Medicinal (side) effects	9 (4.2)	5 (3.5)		4 (5.5)	
Exposure to loud sounds	46 (21.3)	27 (19.0)		19 (26.0)	
Change in hearing	18 (8.3)	9 (6.3)		9 (12.3)	
Sudden deafness	6 (2.8)	3 (2.1)		3 (4.1)	
Changes in air pressure	14 (6.5)	10 (7.0)		4 (5.5)	
Stress/anxiety/depression	14 (6.5)	9 (6.3)		5 (6.8)	
Head/neck trauma	5 (2.3)	2 (1.4)		5 (6.8)	
Jaw problems (TMD)	2 (0.9)	0 (0.0)		2 (2.7)	
Earwax plug	9 (4.2)	4 (2.8)		5 (6.8)	
Fullness/pressure in ears	23 (10.6)	13 (9.2)		10 (13.7)	
Other	16 (7.4)	8 (5.6)		8 (11.0)	
Don’t know	99 (45.8)	74 (52.1)		25 (34.2)	
Missing	2 (0.9)	1 (0.7)		0 (0.0)	
**TFI**^a^					
	16.6 (21.8)	14.7 (19.1)		22.8 (43.1)	1.04 (1.02–1.06)*
N =	212	140		72	
**TFI ranges**					
0–17	109 (50.4)	83 (58.5)		26 (35.6)	Ref
18–31	52 (24.1)	35 (24.6)		17 (23.3)	1.55 (0.75–3.21)
32–53	26 (12.0)	17 (12.0)		9 (12.3)	1.69 (0.67–4.24)
54–72	22 (10.2)	4 (2.8)		18 (24.7)	14.37 (4.46–46.26)*
73–100	3 (1.4)	1 (0.7)		2 (2.7)	6.39 (0.56–73.29)
Missing	4 (1.9)	2 (1.4)		1 (1.4)	
**TFI subscales**^a^					
Intrusiveness	26.7 (32.5)	23.3 (30.0)		40.0 (38.3)	
N =	212	139		73	
Sense of control	43.3 (28.3)	40.0 (22.5)		50.0 (35.0)	
N =	213	140		73	
Cognitive	10.0 (30.0)	6.7 (21.7)		15.0 (47.5)	
N =	211	141		70	
Sleep	10.0 (26.7)	3.3 (20.0)		16.7 (48.3)	
N =	213	140		73	
Auditory	20.0 (49.2)	13.3 (35.0)		30 (56.7)	
N =	212	141		71	
Relaxation	10.0 (26.7)	10.0 (20.0)		18.3 (46.7)	
N =	212	140		72	
Quality of life	2.5 (20.0)	0.0 (15.0)		12.5 (47.5)	
N =	212	140		72	
Emotional	6.7 (20.0)	3.3 (13.3)		20 (41.7)	
N =	213	140		73	

Please see Supplementary Table S2 for answer to location of tinnitus, other; influence of tinnitus, other; potential cause of tinnitus, other.

^a^Median (IQR) *p < 0.05.

^b^Both ears, more in the right ear.

^c^Both ears, more in the left ear.

### Comparison of participants with and without tinnitus

#### Demographic characteristics

Female participants were less likely to have tinnitus compared to male participants (OR 0.60 (95% CI 0.44–0.82) *p* = 0.001) (Table 1). Compared to participants with a low level of education, participants with a higher educational level had higher odds to have tinnitus (OR 1.72 (1.07–2.77) *p* = 0.025) (Table 1).

#### Characteristics on general- and psychological health

Compared to participants without chronic pain, participants with chronic pain were not more likely to have tinnitus (OR 0.87 (95% CI 0.57–1.32), *p* = 0.511). Compared to not having tinnitus, Individuals with a higher score on the HADS-A or the HADS-D did not have higher odds to have tinnitus ((OR HADS-A: 0.99 (95% CI 0.94–1.03) *p* = 0.533, HADS-D (OR 0.99 (95% CI 0.94–1.04) *p* = 0.697).

#### Audiological characteristics and characteristics on noise exposure

The presence of any hearing problem was more frequent in tinnitus participants (135 of 216 (62.5%, 2 missing)) compared to non-tinnitus participants (248 of 690 (36%, 7 missing) (combination of answer options: small-, mediocre-, severe problems and I hear nothing). Compared to participants that did not report any exposure to potentially damaging sound levels, participants with more exposure to potential damaging sound levels had higher odds to have tinnitus multiple times a week but not daily (OR 2.97 (95% CI 1.27–6.92) *p* = 0.012), once a week (OR 2.23 (95% CI 1.04–4.81) *p* = 0.041), less than once a week (OR 1.49 (95% CI 1.05–2.12) *p* = 0.026)) (Table 4).

**Table 4:** Characteristics on general health, psychological health, audiological characteristics and noise and substance behaviour.

Characteristic	Total %	Tinnitus %		OR (95% CI)	Help %		OR (95% CI)
		No	Yes		No	Yes	
**General health**							
**Chronic pain**							
Yes	164 (17.6)	123 (17.8)	35 (16.2)	0.87 (0.57–1.32)	24 (16.9)	11 (15.1)	0.86 (0.39–1.87)
No	745 (79.9)	548 (79.4)	179 (82.9)	Ref	116 (81.7)	62 (84.9)	Ref
Missing	23 (2.5)	19 (2.8)	2 (0.9)		2 (1.4)	0 (0.0)	
**Family history**							
Tinnitus	101 (10.8)	52 (7.5)	43 (19.9)		29 (20.4)	14 (19.2)	
Epilepsy	47 (5.0)	37 (5.4)	10 (4.6)		7 (4.9)	3 (4.1)	
Hearing problem^c^	121 (13.0)	78 (11.3)	37 (17.1)		26 (18.3)	11 (15.1)	
Nerve and/or muscle disease	56 (6.0)	41 (5.9)	13 (6.0)		8 (5.6)	5 (6.8)	
Syndromes	18 (1.9)	15 (2.2)	3 (1.4)		1 (0.7)	2 (2.7)	
Migraines	163 (17.5)	120 (17.4)	40 (18.5)		25 (17.6)	14 (19.2)	
None of the above	538 (57.7)	421 (61.0)	106 (49.1)		68 (47.9)	38 (52.1)	
Missing	23 (2.5)	18 (2.6)	2 (0.9)		2 (1.4)	0 (0.0)	
**General health**							
Excellent	73 (7.8)	57 (8.3)	14 (6.5)		9 (6.3)	5 (6.8)	
Very good	209 (22.4)	170 (24.6)	37 (17.1)		27 (19.0)	9 (12.3)	
Good	492 (52.8)	354 (51.3)	126 (58.3)		82 (57.7)	44 (60.3)	
Fair	133 (14.3)	89 (12.9)	35 (16.2)		22 (15.5)	13 (17.8)	
Bad	6 (0.6)	5 (0.7)	1 (0.5)		0 (0.0)	1 (1.4)	
Missing	19 (2.0)	15 (2.2)	3 (1.4)		2 (1.4)	1 (1.4)	
**Psychological health**							
**HADS-A**^b^							
	3.0 (5.0)	3.0 (4.0)	3.0 (5.0)	0.99 (0.94–1.03)	3.0 (4.0)	4.0 (6.3)	1.11 (1.03–1.20)*
N =	899	667	208		138	70	
**HADS-D**^b^							
	2.0 (4.0)	2 0 (4.0)	1.0 (5.0)	0.99 (0.94–1.04)	1.0 (4.0)	3.0 (5.0)	1.10 (1.02–1.18)*
N =	895	660	211		139	71	
**Psychological health**							
Excellent	183 (19.6)	147 (21.3)	34 (15.7)		23 (16.2)	10 (13.7)	
Very good	289 (31.0)	220 (31.9)	64 (29.6)		48 (33.8)	16 (21.9)	
Good	408 (43.8)	287 (41.6)	103 (47.7)		63 (44.4)	40 (54.8)	
Fair	43 (4.6)	31 (4.5)	12 (5.6)		6 (4.2)	6 (8.2)	
Bad	4 (0.4)	3 (0.4)	1 (0.5)		1 (0.7)	0 (0.0)	
Missing	5 (0.5)	2 (0.3)	2 (0.9)		1 (0.7)	1 (1.4)	
**Audiological**							
**Hyperacusis**							
No. no problem	624 (67.0)	484 (70.1)_	121 (56.0)	Ref	84 (59.2)	36 (49.3)	Ref
Yes. small problem	164 (17.6)	115 (16.7)	46 (21.3)	1.60 (1.08–2.38)*	33 (23.2)	13 (17.8)	0.92 (0.43–1.95)
Yes. mediocre problem	99 (10.6)	60 (8.7)	37 (17.1)	2.47 (1.56–3.89)*	19 (13.4)	18 (24.7)	2.21 (1.04–4.70)*
Yes. large problem	30 (3.2)	22 (3.2)	8 (3.7)	1.46 (0.63–3.35)	4 (2.8)	4 (5.5)	2.33 (0.55–9.85)
Yes very large problem	3 (0.3)	1 (0.1)	1 (0.5)	4.0 (0.25–64.41)	0 (0.0)	1 (1.4)	Error
Missing	12 (1.3)	8 (1,2)	3 (1.4)		2 (1.4)	1 (1.4)	
**Hearing problems**							
Yes. I hear nothing	11 (1.2)	4 (0.6)	4 (1.9)	5.51 (1.35–22.47)*	1 (0.7)	3 (4.1)	10.77 (1.05–110.21)*
Yes. severe problems	42 (4.5)	29 (4.2)	13 (6.0)	2.47 (1.23–4.95)*	6 (4.2)	7 (9.6)	4.19 (1.24–14.12)*
Yes. mediocre problems	110 (11.8)	56 (8.1)	47 (21.8)	4.62 (2.93–7.29)*	23 (16.2)	24 (32.9)	3.74 (1.71–8.21)*
Yes. small problems	235 (25.2)	159 (23.0)	71 (32.9)	2.46 (1.70–3.55)*	49 (34.5)	22 (30.1)	1.61 (0.77–3.36)
No. no problems	524 (56.2)	435 (63.0)	79 (36.6)	Ref	61 (43.0)	17 (23.3)	Ref
Missing	10 (1.1)	7 (1.0)	2 (0.9)		2 (1.4)	0 (0.0)	
**Use of**							
Hearing aid	120 (12.9)	74 (10.7)	40 (18.5)	1.93 (1.27–2.94)*	18 (12.7)	22 (30.1)	3.15 (1.55–6.39)*
Cochlear Implant	4 (0.4)	2 (0.3)	1 (0.5)	1.78 (016–19.79)	0 (0.0)	1 (1.4)	Error
Sound generator / Tinnitus Masker	3 (0.3)	1 (0.1)	2 (0.9)	7.14 (0.64–79.18)	0 (0.0)	2 (2.7)	Error
Combination (hearing aid + masker)	2 (0.2)	1 (0.1)	1 (0.5)	3.57 (0.22–57.34)	0 (0.0)	1 (1.4)	Error
None	790 (84.8)	603 (87.4)	169 (78.2)	Ref	121 (85.2)	47 (64.4)	Ref
Missing	13 (1.4)	9 (1.3)	3 (1.4)		3 (2.1)	0 (0.0)	
**Auditory hallucinations**							
No	842 (90.3)	637 (92.3)	183 (84.7)	0.46 (0.28–0.76)*^a^	123 (86.6)	59 (80.8)	0.60 (0.26–1.36)^a^
Yes. Understandable voices	9 (1.0)	4 (0.6)	5 (2.3)		2 (1.4)	3 (4.1)	
Yes. Not understandable voices	19 (2.0)	11 (1.6)	7 (3.2)		4 (2.8)	3 (4.1)	
Yes. Music	24 (2.6)	14 (2.0)	10 (4.6)		5 (3.5)	5 (6.8)	
Yes. Telephone. Doorbell. Alarm. Sirens	33 (3.5)	19 (2.8)	12 (5.6)		8 (5.6)	4 (5.5)	
Yes. Footsteps	4 (0.4)	3 (0.4)	1 (0.5)		1 (0.7)	0 (0.0)	
Yes. Machines or vehicles	6 (0.6)	2 (0.3)	4 (1.9)		2 (1,4)	2 (2.7)	
Yes. Animals	8 (0.9)	4 (0.6)	3 (1.4)		2 (1.4)	1 (1.4)	
Yes. Other	6 (0.6)	4 (0.6)	2 (0.9)		1 (0.7)	1 (1.4)	
Missing	18 (1.9)	10 (1.4)	6 (2.8)		4 (2.8)	2 (2.7)	
**Noise and substance behaviour**							
**Use of head/ ear phones**							
No	550 (59.0)	416 (60.3)	115 (53.2)	Ref	80 (56.3)	34 (46.6)	Ref
Less than once a week	162 (17.4)	123 (17.8)	37 (17.1)	1.09 (0.71–1.66)	22 (15.5)	15 (20.5)	1.60 (0.74–3.46)
Once a week	52 (5.6)	34 (4.9)	17 (7.9)	1.81 (0.98–3.36)	12 (8.5)	5 (6.8)	0.98 (0.32–2.998)
Multiple times a week	106 (11.4)	74 (10.7)	31 (14.4)	1.52 (0.95–2.42)	18 (12.7)	13 (17.8)	1.70 (0.75–3.85)
Daily	50 (5.4)	35 (5.1)	14 (6.5)	1.45 (0.75–2.78)	8 (5.6)	6 (8.2)	1.77 (0.57–5.47)
Missing	12 (1.3)	8 (1.2)	2 (0.9)		2 (1.4)	0 (0.0)	
**Potential damaging sound levels**							
No	620 (66.5)	478 (69.3)	124 (57.4)	Ref	78 (54.9)	45 (61.6)	Ref
Daily	14 (1.5)	8 (1.2)	5 (2.3)	2.41 (0.78–7.49)_	3 (2.1)	2 (2.7)	1.16 (0.19–7.18)
Multiple times a week	24 (2.6)	13 (1.9)	10 (4.6)	2.97 (1.27–6.92)*	8 (5.6)	2 (2.7)	0.43 (0.09–2.13)
Once a week	30 (3.2)	19 (2.8)	11 (5.1)	2.23 (1.04–4.81)*	6 (4.2)	5 (6.8)	1.44 (0.42–5.00)
Less than once a week	230 (24.7)	163 (23.6)	63 (29.2)	1.49 (1.05–2.12)*	44 (31.0)	19 (26.0)	0.75 (0.39–1.44)
Missing	14 (1.5)	9 (1.3)	3 (1.4)		3 (2.1)	0 (0.0)	
**If yes. use of hearing protection (n = 298)**							
Never	157 (52.7)	118 (58.1)	35 (39.3)	Ref	23 (37.7)	12 (42.9)	Ref
Sometimes	87 (29.2)	48 (23.6)	38 (42.7)	2.67 (1.51–4.71)*	25 (41.0)	13 (46.4)	0.997 (0.38–2.62)
Often	32 (10.7)	20 (9.9)	12 (13.5)	2.02 (0.90–4.54)	9 (14.8)	3 (10.7)	0.64 (0.15–2.81)
Always	22 (7.4)	17 (8.4)	4 (4.5)	0.79 (0.25–2.51)	4 (6.6)	0 (0.0)	Error
Missing	0 (0.0)	0 (0.0)	0 (0.0)		0 (0.0)	0 (0.0)	
**Smoker**							
Never	346 (37.1)	266 (38.6)	74 (34.3)		45 (31.7)	29 (39.7)	
At this moment	68 (7.3)	54 (7.8)	14 (6.5)		8 (5.6)	5 (6.8)	
Used to smoke	505 (54.2)	362 (52.5)	125 (57.9)		87 (61.3)	38 (52.1)	
Missing	13 (1.4)	8 (1.2)	3 (1.4)		2 (1.4)	1 (1.4)	
**Drug use**							
Never	881 (94.5)	658 (95.4)	199 (92.1)		130 (91.5)	68 (93.2)	
Used to	22 (2.4)	15 (2.2)	7 (3.2)		4 (2.8)	3 (4.1)	
Sometimes	9 (1.0)	5 (0.7)	4 (1.9)		4 (2.8)	0 (0.0)	
Regularly	4 (0.4)	2 (0.3)	2 (0.9)		1 (0.7)	1 (1.4)	
Missing	16 (1.7)	10 (1.4)	4 (1.9)		3 (2.1)	1 (1.4)	
Average # of glasses alcohol a week^b^	2 (7)	2 (7)	2 (7)		3 (7)	2 (7)	
N =	888	660	205		134	70	

Please see Supplementary Table S2 for answer to auditory hallucinations, other.

^a^Reference is yes.

^b^Median (IQR) *p < 0.05.

^c^Hearing problem for which hearing aids were used before 60th year of age.

### Help seeking participants

Of the 216 tinnitus participants, 72 (1 missing, 33.3%) had sought help for their tinnitus. Of the remaining 143 of 216 (66.2%, 1 missing), one (0.7%, 2 missing) planned to seek help. We defined 73 of 216 tinnitus participants (33.8%, 1 missing), as a help seeking tinnitus participant, and 142 of 216 (65.7% 1 missing) as non-help seeking tinnitus participants. Most help seekers were treated or planned treatment at a doctor (39 of 73, (53.4% 9 missing)), followed by audiological care (21 of 73, (28.8% 9 missing)) (Table 2).

### Comparison of help seekers versus non-help seekers

#### Demographics

Twenty-six of 73 help seekers (HS) were female (35.6%, 0 missing), compared to 66 of 142 (46.5%, 0 missing) of non-help seekers (NHS). Compared to males, females were not more likely to seek help for tinnitus (OR 0.64 (95% CI 0.36–1.14) *p* = 0.129). The help-seekers had a median age of 69 (IQR 13) years, compared to 66.0 (IQR 16) years of age in the in the non-help seekers (Table 1).

#### Tinnitus characteristics

Help seeking tinnitus participants more often considered their tinnitus to be a reasonable (23 out of 73 (31.5%)) or a large problem (8 of 73 (11.0%)), compared to the non-help seekers ((respectively 20 of 142 (14.1%, 0 missing) (OR 18.4 (95% CI 4.96–68.29), *p* = 0.000) and (4 of 142 (2.8%, 0 missing) (OR 32.0 (6.0–170.6), *p* = 0.000))). Individuals with a higher TFI score were more prone to seek help,, compared to not seek help (OR 1.04 (95% CI 1.02–1.06), *p* = 0.000) (Table 3). Twenty-three of 73 (31.5%, 0 missing) of the help seekers experienced more than one sound, compared to 25 of 142 (17.6, 0 missing) of the non-help seekers. The experience of a constant tinnitus pattern compared to an intermittent pattern increased the odds of seeking help (OR 3.26 (95% CI 1.69–6.30) *p* = 0.000). A varying tinnitus loudness compared to a non-varying loudness increased the odds of seeking help (OR 2.97 (95% CI 1.62–5.46) *p* = 0.000).

#### Characteristics on general- and psychological health

In participants with or without chronic pain the odds for seeking help were equal (OR 0.86 (95% CI 0.39–1.87), *p* = 0.698). Participants with higher HADS-A scores or HADS-D scores were more likely to seek help ((HADS-A OR 1.11 (1.03–1.20), *p* = 0.011), (HADS-D OR 1.10 (95% CI 1.02–1.18), *p* = 0.012)) (Table 4). Compared to non-help seekers, help seekers had higher percentages of the following diseases diagnosed by a physician: dental problems (HS: 13 of 73, (17.8%, 0 missing), NHS: 11 of 142, (7.7%, 6 missing), depression (HS: 10 of 73, (13.7%, 0 missing), NHS: 5 of 142, (3.5%, 6 missing)), balance problems/vertigo (HS: 13 of 73, (17.8%, 0 missing), NHS: 9 of 142, (6.3%, 6 missing)) and hearing loss (HS: 26 of 73, (35.6%, 0 missing), NHS: 25 of 142 (17.6%, 6 missing)). (Supplementary Table S1).

#### Audiological characteristics and characteristics on noise exposure

Participants who judged sounds as a mediocre problem (hyperacusis) were more likely to seek help for tinnitus than not to seek help for their tinnitus (HS: 18 of 73 (24.7%, 1 missing), NHS: 19 of 142 (13.4%, 2 missing)) compared to ‘no problem’ (OR 2.21 (95% CI 1.04–4.70), *p* = 0.039)). The subjective presence of any hearing problem was more frequent in help seeking tinnitus participants (HS: 56 of 73 (76.7%, 0 missing) versus non-help seeking participants (79 of 142 (55.6%, 2 missing) (combination of answer options: small-, mediocre-, severe problems and I hear nothing) (Table 4).

Participants that had exposed themselves to potential damaging sound levels were not more likely to seek help compared to not seek help (reference: no exposure to damaging sound levels, daily: (OR 1.16 (95% CI 0.19–7.18) *p* = 0.88), multiple times a week: (OR 0.43 (95% CI 0.09–2.13) *p* = 0.43), once a week: (OR 1.44 (95% CI 0.42–5.00) *p* = 0.56), less than once a week: (OR 0.75 (95% CI 0.39–1.44) *p* = 0.38).

## Discussion

In this study our primary objective was to describe differences in characteristics of help seeking versus non-help seeking tinnitus participants by means of a questionnaire. It was sent to an adult sample of inhabitants of the Netherlands.

Help seeking tinnitus participants had a higher median score on the TFI compared to non-help seeking tinnitus participants. These numbers illustrate that a higher distress score is more frequent in individuals who seek help. We defined participants as a help seeking participant when they planned to seek help for their tinnitus within the next month or had already sought help. We added no time limitations on how long ago in the past they sought help to this definition. Consequently, people could had already sought help years ago, and did not have an active wish for help at the moment of the questionnaire. Interestingly, the help seeking group consisted for 99% (72 of 73) out of participants that had already sought help for their tinnitus. Even though their initial tinnitus distress levels might have been higher, people were still experiencing a median score of 22.8 on the TFI, which indicates they consider their tinnitus to be a ‘’small problem’’ even after seeking help in the past^22^. Besides this, several questions regarding tinnitus remain; what makes people transit from ‘having’ tinnitus towards becoming a tinnitus patient or having tinnitus disorder^4^? 35.6% of the help seekers, as identified in our study, had a TFI score ranging between 0 and 17, which can be interpreted as ‘’not a problem’’^17^. This might illustrate the controversies between experienced distress scores by these validated instruments and the willingness/need of people to seek help.

We found an overlap in known risk factors for tinnitus in literature, with higher frequencies in help seekers^23^. This is to be expected since many studies that assessed tinnitus risk factors were performed in a hospital population of people with tinnitus. These samples include help seekers by definition. For example, hyperacusis and hearing loss were more common in those with tinnitus that sought help compared to those with tinnitus that did not sought help. These two are also two known risk factors for tinnitus and tinnitus distress in literature^7,23,24^.

Surprisingly, we did not find a statistically significant difference for age in help seekers and non-help seekers. Especially since advanced age is a risk factors of tinnitus^23^. We believe this might be caused by the advanced, and reasonably low variance in age of the complete sample.

In our study, we found no clinically relevant differences in anxiety or depression scores measured by the HADS between help seekers and non-help seekers. Even though the odds of having a higher score on both the anxiety and depression scale were significantly higher in the help seeking tinnitus group in our study, the median scores on both scales were all below eight. A score below eight score does not indicate a possible depression or anxiety^20^. We therefore believe that these statistically significant results are not clinically relevant. However, we did find higher frequencies of a self-reported clinical diagnosis of depression in those that had sought help. This discrepancy might be caused by the difference in timing of both questions. The HADS assesses depression or anxiety at the moment of filling out the survey. A clinical diagnosis of depression might have been made years ago. We know from literature that depression is a common risk factor for tinnitus, and was also one of the four most important variables for tinnitus subtyping^6^. The low scores on the HADS might be caused by the fact that our survey was distributed among a general population sample, rather than a hospital sample. The low scores are comparable to a population study from Norway describing similar outcomes in people with and without tinnitus^25^.

With respect to tinnitus specific characteristics, we found that 31.5% of the help seekers experience more than one sound, compared to 17.6% in the non-help seeking group. We also found help seekers to experience a varying loudness more often (65.8%) compared to those that do not seek help (40.8%). This is comparable to a study by Lilllemor et al. from 1993 in a hospital setting. They reported ‘’complainers’’ to hear more than one sound. However, contrasting to our study they report a non-fluctuating sound to be heard by complainers more often than ‘’non-complainers’’^10^. These differences in characteristics could point out the way people cope with their tinnitus. One could hypothesize that varying loudness or several sounds make tinnitus more difficult to cope with.

### Strengths and weaknesses

A strength of the presented study is the large quantity of data regarding tinnitus and individual characteristics, collected from a sample from the general Dutch population. We created unique data about people with tinnitus that seek help versus those that do not. There are several limitations applicable to this study. The first is that, while the study was set out in a sample of the Dutch population, in terms of age the individuals that responded were not representative of the Dutch population^26^. This might be due to the fact only panel members who gave permission to combine their answers of the survey with health care consumption data as registered by their general practitioner were invited for the survey^13^. The lack of representability may also partly due to the response rate of 41.4%. The response rate might have been influenced by the lengthiness of the questionnaire (with a maximum of 8 pages) or the topic of the questionnaire. This could have made people with tinnitus more inclined to fill out the questionnaire. Due to space limitations we had to take decisions on which questions to include. Still, we did include a validated tinnitus distress measures (the TFI) and a validated anxiety and depression measure to assess these variables of importance for subtyping^6,18,19,22^. Another limitation is our definition of tinnitus. We based it on frequency and duration, but tinnitus distress was not included in our definition.

### Future perspectives

Tinnitus heterogeneity is one of the main impediments that hinder the search for a curative tinnitus treatment^27^. The presented outcomes might help to gain insight in the issue of heterogeneity. However, we believe that the only way to succeed in disentangling this heterogeneity, possibly with subtypes or prediction models, is with interdisciplinary and collaborative research with sound methodology and large datasets^3^. The first steps in multidisciplinary cooperation in research as well as training have been taken, such as programs like ESIT, Tinnitus Assessment Causes Treatment (TINACT) and Unification of Treatments and Interventions for Tinnitus Patients (UNITI)^26–30^.

## Conclusions

This study pioneered in describing individual characteristics in the general population between people with tinnitus that sought help versus those who did not. Differences between groups were mainly identified in tinnitus characteristics and audiological characteristics. The outcomes of this study could serve as an initial step to detangle the heterogeneity in tinnitus patients.

## Supplementary Information

Supplementary Methods S1.

Supplementary Methods S2.

Supplementary Table S1.

Supplementary Table S2.

## Data Availability

The datasets presented in this article are not readily available because the Dutch Health Care Consumer Panel has a program committee, which supervises processing the data of the Dutch Health Care Consumer Panel and decides about the use of the data. This program committee consists of representatives of the Dutch Ministry of Health, Welfare and Sport, the Health Care Inspectorate, Zorgverzekeraars Nederland (Association of Health Care Insurers in the Netherlands), the National Health Care Institute, the Federation of Patients and Consumer Organisations in the Netherlands, the Dutch Healthcare Authority and the Dutch Consumers Association. All research conducted within the Consumer Panel has to be approved by this program committee. The committee assesses whether a specific research fits within the aim of the Consumer Panel, that is strengthened by the position of the health care user. Requests to access the datasets should be directed to the corresponding author.
