# Supplementary material for: Differences in characteristics between people with tinnitus that seek help and that do not
Source: Sci Rep. 2021 Nov 25;11:22949. doi: 10.1038/s41598-021-01632-5 (PMC8616930; doi:10.1038/s41598-021-01632-5)
Supplement: Supplementary file 2 — Supplementary Methods S2. [file 41598_2021_1632_MOESM2_ESM.pdf]

Supplementary Methods S2

Please note, this questionnaire was freely translated from Dutch to English for the purpose of providing the reader with more information on the questions. The translations were not validated.

A. Background

1. What is your date of birth? (day-month-year) ☐☐ ☐☐ ☐☐☐☐

2. What is your gender?
   - ☐ Male
   - ☐ Female

B. Tinnitus

We would like to know how many people in the Netherlands suffer from tinnitus. Tinnitus is the hearing of e.g. a beep, whistle, sis, zoom or another sound without the actual presence of the sound in your surroundings.

3. Did you experience tinnitus in the last year? Tinnitus is the hearing of e.g. a beep, whistle, sissing, zoom or another sound without the actual presence of the sound in your surroundings This can last a very short amount of time or a whole day.
   - ☐ No → ***go to question 38 (part D)***
   - ☐ Yes

4. If you experience tinnitus, how long does the tinnitus last?
   - ☐ Less than 5 minutes → ***go to question 5***
   - ☐ Between 5-60 minutes → ***go to question 6***
   - ☐ 60 minutes or more → ***go to question 7***
   - ☐ Continuous (the entire day) → ***go to question 7***

5. How often do you experience tinnitus?
   - ☐ Daily or almost daily
   - ☐ Weekly
   - ☐ Monthly
   - ☐ Once or less than once a year

→ ***go to question 38***

6. How often do you experience tinnitus?
   - ☐ Daily or almost daily → ***go to question 8***
   - ☐ Weekly → ***go to question 8***
   - ☐ Monthly → ***go to question 38***
   - ☐ Once or less than once a year → ***go to question 38***

7. How often do you experience tinnitus?
   - ☐ Daily or almost daily
   - ☐ Weekly
   - ☐ Monthly
   - ☐ Once or less than once a year → ***go to question 38***

8. Which pattern best describes your tinnitus during the day?

☐ Continuously: you can hear it all the time or most of the time
☐ Intermittently: it comes and goes

9. When did your tinnitus start?
   ☐ Less than 3 months ago
   ☐ 3 to 6 months ago
   ☐ 6 months ago ore more

10. How big a problem is your tinnitus at this moment?
   ☐ No problem
   ☐ Small problem
   ☐ Reasonable problem
   ☐ Large problem
   ☐ Very large problem

11. Have you every sought help for you tinnitus? (E.g. through the internet, caregiver or physician)
   ☐ No
   ☐ Yes → ***go to question 13***

12. Are you planning to seek help for your tinnitus within now and a month?
   ☐ No → ***go to question 14***
   ☐ Yes

13. Have you ever been treated, are you currently being treated, or are you planning on getting treated with one of the next treatment for your tinnitus? You can choose multiple options.
   ☐ Psychiatric treatment
   ☐ Psychological treatment (for example Cognitive Behavioral Therapy (CBT), Tinnitus Retraining Therapy (TRT), Mindfulness)
   ☐ Audiological treatment (for example hearing aids, tinnitus maskers)
   ☐ Physiotherapy (for example manual therapist)
   ☐ Self-management (for example nutritional supplement / medicinal herbs, self-help books)
   ☐ Alternative therapy (for example acupuncture, chiropractor, homeopathic supplies, yoga, haptotherapist)
   ☐ Visit to a physician
   ☐ Other, namely:

14. How intrusive is your tinnitus at this moment?

Totally not intrusive *Extremely intrusive*

0	1	2	3	4	5	6	7	8	9	10
☐	☐	☐	☐	☐	☐	☐	☐	☐	☐	☐

15. Do you hear one type or more sounds?
   ☐ One type of sound
   ☐ Different types of sounds

In the case you hear more than one type of sound, please choose the answer in the next questions that best describes the most bothering sound.

16. Is your tinnitus pulsatile (for example with the heartbeat)?
    - ☐ No
    - ☐ Yes

17. Where do you experience your tinnitus? (You can choose multiple options)
    - ☐ Right ear
    - ☐ Left ear
    - ☐ Both ears, mostly right
    - ☐ Both ears, mostly left
    - ☐ Both ears equally
    - ☐ Inside my head
    - ☐ Other, namely: ____________

18. How was the start of your tinnitus?
    - ☐ Gradually
    - ☐ Suddenly

19. Does the loudness of your tinnitus vary over time?
    - ☐ No
    - ☐ Yes

20. What is the pitch of your tinnitus like?
    - ☐ High
    - ☐ Average
    - ☐ Low
    - ☐ I don't know

21. Is your tinnitus positively and/or negatively influenced by any of the options below? *(*You can choose multiple options*)*
    - ☐ Presence of a loud sound
    - ☐ Music or particular ambient noise? (like the sound of a waterfall)
    - ☐ Head or neck movements (for example moving the jaw forwards, or clamping the teeth)
    - ☐ If your arms/neck touch your head.
    - ☐ Sleeping during the day
    - ☐ Good quality of sleep
    - ☐ Stress
    - ☐ Medicines
    - ☐ The use of hearing aids
    - ☐ Other, namely: ____________
    - ☐ My tinnitus is not influenced by anything

22. Was the start of your tinnitus related to? (You can choose multiple options*)*
    - ☐ Flu, cold or another infection
    - ☐ (side)effect of medicine

- ☐ Exposure to loud sounds
- ☐ Change in hearing (not sudden deafness)
- ☐ Sudden deafness
- ☐ Exposure to changes in air pressure (for example in an airplane or during scuba diving)
- ☐ Stress, anxiety or depression
- ☐ Head trauma / neck trauma (for example whiplash)
- ☐ Jaw problem (TMD)
- ☐ Earwax plug
- ☐ The feeling of fullness of the ears or pressure in the ears.
- ☐ Other, namely:

- ☐ I don’t know

C. Tinnitus distress

Meikle, M. B. et al. The tinnitus functional index: development of a new clinical measure for chronic, intrusive tinnitus.[Erratum appears in Ear Hear. 2012 May;33(3):443]. Ear Hear. 33, 153–176 (2012).

Tromp R. De betrouwbaarheid en validiteit van de Nederlandstalige versie van de Tinnitus Functional Index (TFI). *Univ Med Cent Groningen.* (2014). Masters.

D. Mood (start question 38)

Spinhoven, P. et al. A validation study of the Hospital Anxiety and Depression Scale (HADS) in different groups of Dutch subjects. Psychol. Med. 27, 363–370 (1997).

Zigmond AS, S. R. The hospital anxiety and depression scale. Acta Psychiatr Scand 67, 361–370 (1983).

E. Questions regarding sounds, hearing and general health.

52. Have sounds been a problem for you in the last week? Sounds that were too loud or uncomfortable to you, whilst these seemed normal to others around you? *Please note, we mean all sounds other than tinnitus.*

- ☐ No, no problem
- ☐ Yes, a small problem
- ☐ Yes, a mediocre problem
- ☐ Yes, a large problem
- ☐ Yes, a very large problem

53. Do you experience problems with hearing, without using a hearing aid or another hearing tool?

- ☐ Yes, i hear nothing
- ☐ Yes, severe problems
- ☐ Yes, mediocre problems

☐ Yes, small problems
☐ No, no problem

54. Do you use one or more of the next machines? *(You can choose multiple options)*

☐ Hearing aids
☐ Cochlear implant
☐ Sound generator / Tinnitus masker
☐ A combination (hearing aid and sound generator within one machine)
☐ No

55. Do you experience chronic pain? (more than 6 months)

☐ No
☐ Yes

56. Tick the boxes if the next diseases / conditions occur in your family. With family we mean biological brothers/sisters, (grand)parents, uncles/aunts, (grand)children. *(You can choose multiple options)*

☐ Tinnitus (tinnitus)
☐ Epilepsy
☐ Hearing problem, with use of hearing aids before the 60th year of age.
☐ Nerve and/or muscle disease
☐ Syndromes
☐ Migraines
☐ None of these diseases / conditions.

57. Do you ever listen to sounds (for example music) through headphones or earphones?

☐ No
☐ Yes, less than once a week
☐ Yes, once a week
☐ Yes, multiple times a week but not daily
☐ Yes, daily

58. Do you ever expose yourself to potential harmful sound levels? (for example loud music in a pub, during your work (construction), shooting)

☐ No ➔ ***go to question 60***
☐ Yes, daily
☐ Yes, multiple times a week but not daily
☐ Yes, once a week
☐ Yes, less than once a week

59. How often do you wear hearing protection?

☐ Never
☐ Sometimes
☐ Often
☐ Always

60. Which of the following descriptions best suits your smoking behavior?

☐ I have never smoked
☐ I smoke at the moment
☐ I used to smoke

61. What is the average amount of glasses of alcohol you drink weekly? ☐

62. Which of the following descriptions best suits your drug use?
☐ I have never used drugs
☐ I used to use drugs
☐ I sometimes use drugs
☐ I use drugs on a regular basis

63. Which of the conditions/disease below has a physician diagnosed you with? *You can choose multiple options.*
☐ Temporomandibular (jaw (joint)) pain (TMD)
☐ Dental problems
☐ Sleeping disorder
☐ Meningitis
☐ Multiple sclerosis (MS)
☐ Epilepsy
☐ Stroke
☐ Anxiety
☐ Depression
☐ Emotional trauma
☐ Excessive stress
☐ High blood pressure
☐ Heart attack
☐ Chronic fatigue syndrome
☐ Thyroid conditions
☐ Diabetes
☐ Hyperinsulinemia
☐ High cholesterol
☐ Rheumatoid arthritis
☐ Systemic lupus erythematosus (SLE)
☐ Chronic sinusitis
☐ Balance or vertigo problems
☐ Recurring ear infections
☐ Hearing loss
☐ Anemia
☐ Heartburn / gastroesophageal reflux
☐ Globus (lump in the throat)
☐ Other, namely:
☐ None of these conditions/diseases

64. Does it ever happen that you hear someone speaking, whilst nobody is there? Sounds or music can also be heard, while it is unclear where it comes from. (You can choose multiple options)
☐ No
☐ Yes, understandable voices
☐ Yes, not understandable voices
☐ Yes, music
☐ Yes, telephone/doorbell/alarm/sirens
☐ Yes, footsteps
☐ Yes, vehicles or machines
☐ Yes, other
☐ Yes, Other, namely:
