# Supplementary material for: Differences in characteristics between people with tinnitus that seek help and that do not
Source: Sci Rep. 2021 Nov 25;11:22949. doi: 10.1038/s41598-021-01632-5 (PMC8616930; doi:10.1038/s41598-021-01632-5)
Supplement: Supplementary file 3 — Supplementary Table S1. [file 41598_2021_1632_MOESM3_ESM.docx]

Supplementary Table S1. Diseases diagnosed by a physician. ^1^TMD = Temporomandibular dysfunction *G.E.R.D = gastroesophageal reflux disease

| **Disease** | **Total**  **n (%)** | | **Tinnitus n (%)** | | | **Help-seeker n (%)** | | |  |
| --- | --- | --- | --- | --- | --- | --- | --- | --- | --- |
|  |  | | **No** | **Yes** | | **No** | **Yes** | |  |
| TMD^1^ | 9 (1) | | 6 (0.9) | 3 (1.4) | | 0 (0.0) | 3 (4.1) | |  |
| Dental problems | 70 (7.5) | | 46 (6.7) | 24 (11.1) | | 11 (7.7) | 13 (17.8) | |  |
| Sleeping disorder | 52 (5.6) | | 33 (4.8) | 15 (6.9) | | 9 (6.3) | 6 (8.2) | |  |
| Meningitis | 10 (1.1) | | 7 (1.0) | 3 (1.4) | | 0 (0.0) | 3(4.1) | |  |
| Multiple sclerosis | 2 (0.2) | | 2 (0.3) | 0 (0.0) | | 0 (0.0) | 0 (0.0) | |  |
| Epilepsy | 7 (0.8) | | 6 (0.9) | 1 (0.5) | | 1 (0.7) | 0 (0.0) | |  |
| Stroke | 27 (2.9) | | 23 (3.3) | 4 (1.9) | | 3 (2.1) | 1 (1.4) | |  |
| Anxiety | 21 (2.3) | | 16 (2.3) | 5 (2.3) | | 3 (2.1) | 2 (2.7) | |  |
| Depression | 50 (5.4) | | 33 (4.8) | 15 (6.9) | | 5 (3.5) | 10 (13.7) | |  |
| Emotional Trauma | 25 (2.7) | | 18 (2.6) | 7 (3.2) | | 4 (2.8) | 3 (4.1) | |  |
| Excessive stress | 7 (0.8) | | 5 (0.7) | 2 (0.9) | | 2 (1.4) | 0 (0.0) | |  |
| High blood pressure | 260 (27.9) | | 181(26.2) | 66 (30.6) | | 44 (31.0) | 22 (30.1) | |  |
| Myocardial infarct | 45 (4.8) | | 30 (4.3) | 14 (6.7) | | 5 (3.5) | 9 (12.3) | |  |
| Chronic fatigue | 7 (0.8) | | 2 (0.3) | 4 (1.9) | | 2 (1.4) | 2 (2.7) | |  |
| Thyroid problems | 50 (5.4) | | 41 (5.9) | 9 (4.2) | | 7 (4.9) | 2 (2.7) | |  |
| Diabetes | 77 (8.3) | | 55 (8.0) | 16 (7.4) | | 9 (6.3) | 7 (9.6) | |  |
| Hyperinsulinemia | 0 (0.0) | | 0 (0.0) | 0 (0.0) | | 0 (0.0) | 0 (0.0) | |  |
| High cholesterol | 199 (21.4) | | 151 (21.9) | 41 (19.0) | | 26 (18.3) | 15 (20.5) | |  |
| Rheumatoid arthritis | 47 (5.0) | | 34 (4.9) | 12 (5.6) | | 7 (4.9) | 5 (6.8) | |  |
| SLE | 2 (0.2) | | 2 (0.3) | 0 (0.0) | | 0 (0.0) | 0 (0.0) | |  |
| Chronic sinusitis | 14 (1.5) | | 12 (1.7) | 2 (0.9) | | 2 (1.4) | 0 (0.0) | |  |
| Balance problems / vertigo | 67 (7.2) | | 42 (6.1) | 22 (10.2) | | 9 (6.3) | 13 (17.8) | |  |
| Recurrent ear infections | 18 (1.9) | | 13 (1.9) | 5 (2.3) | | 3 (2.1) | 2 (2.7) | |  |
| Hearing loss | 140 (15.0) | | 81 (11.7) | 51 (23.6) | | 25 (17.6) | 26 (35.6) | |  |
| Anemia | 30 (3.2) | | 21 (3.0) | 8 (3.7) | | 5 (3.5) | 3 (4.1) | |  |
| G.E.R.D.* | 24 (2.6) | | 18 (2.6) | 6 (2.8) | | 6 (4.2) | 0 (0.0) | |  |
| Globus | 7 (0.8) | | 3 (0.4) | 3 (1.4) | | 2 (1.4) | 1 (1.4) | |  |
| None | 245 (26.3) | | 191 (27.7) | 51 (23.6) | | 36 (25.4) | 14 (19.2) | |  |
| Missing | 23 (2.5) | | 15 (2.2) | 6 (2.8) | | 6 (4.2) | 0 (0.0) | |  |
| Other | 218 (30.9) | | | | | | | | |
| *Tractus Digestivus* | | 19 | | | *Malignancies* | | | 19 | |
| *Morbus Bechterew* | | 4 | | | *Pulmonary diseases* | | | 25 | |
| *Cardiac disease* | | 22 | | | *Fibromyalgia* | | | 8 | |
| *Diseases of the musculoskeletal system* | | 54 | | | *Gallbladder problems* | | | 4 | |
| *Benign prostate diseases* | | 5 | | | *Neurological diseases* | | | 20 | |
| *Psychiatric diseases* | | 6 | | | *Morbus Meniere* | | | 2 | |
| *Vascular diseases* | | 10 | | | *Gynecological diseases* | | | 3 | |
| *Skin diseases* | | 6 | | | *Eye diseases* | | | 8 | |
| *Osteoporosis/penia* | | 4 | | | *Kidney / urological diseases* | | | 4 | |
| *Headache / migraine* | | 11 | | | *Other* | | | 47 | |
