# Supplementary material for: Differences in characteristics between people with tinnitus that seek help and that do not
Source: Sci Rep. 2021 Nov 25;11:22949. doi: 10.1038/s41598-021-01632-5 (PMC8616930; doi:10.1038/s41598-021-01632-5)
Supplement: Supplementary file 4 — Supplementary Table S2. [file 41598_2021_1632_MOESM4_ESM.docx]

Supplementary Table S2. Answers to other, namely.

| **Characteristic** | **Answer to other, namely** |
| --- | --- |
|  |  |
| Type of tinnitus help  (n = 7) | *No, no treatment* |
|  | *Cannot be treated* |
|  | *I learned to live with it. I have tinnitus in both ears. Also, I constantly have a different melody in one or both ears. So sometimes four sounds mixed together. Luckily I am quite deaf so that little noise comes from outside.* |
|  | *No* |
|  | *No* |
|  | *Cochlear implant* |
|  | *Accept that you have it and learn to live with it yourself. If you focus your attention to something else you hear it less* |
|  |  |
|  |  |
| Location of tinnitus (n = 2) | *A whiz in both ears and outside my head (mostly to the right of my head I hear different melodies blabla)* |
|  | *I can only hear it during the night when everything is silent, I hear a disturbing sound from way way. It seems like a stationary running car.* |
|  |  |
| Influence of tinnitus (n = 22) | *Use of salt, a loud whiz with a lot of salt* |
|  | *Lying down* |
|  | *Lyme* |
|  | *When there is a lot of noise, I do not hear the beep any more* |
|  | *Mostly when getting up* |
|  | *Changes in air pressure, when I put my head on the pillow and lie on an ear, bending down. In combination with migraines, balance disorder* |
|  | *Whiz is always presence, but I am only aware of it when I notice it (like now). It sometimes gets worse with a cold / the flu.* |
|  | *Silence around me* |
|  | *In the evening* |
|  | *I try not to notice it* |
|  | *Comes and goes, it gets stronger when I think about it* |
|  | *In rest before I go to sleep I put a finger to / inside my ear. Very stupid.* |
|  | *Yawning* |
|  | *Extra distress when there is a monotonous sound or whiz or hum* |
|  | *It gets worse with fatigue* |
|  | *I hear it the most in silence (in bed, before falling asleep)* |
|  | *With a rhinitis* |
|  | *With exercise, e.g. taking the stairs* |
|  | *When I shift focus (work/book) I hear them less loudly. Especially difficult when I wake up at night and I hear one of the most irritating melodies. I cannot always ignore them and* |
|  | *When I give it attention, I hear it constantly. Distraction helps.* |
|  | *Absence of sound* |
|  | *Distraction* |
|  |  |
| Potential cause of tinnitus (n = 16) | *Probably high blood pressure* |
|  | *Silence* |
|  | *After a cerebral hemorrhage* |
|  | *After the 4^th^ operation above my right eye (resection meningioma right frontotemporal)* |
|  | *Migraine / tension headache. Dizziness.* |
|  | *Left also a perforated eardrum, influence unknown* |
|  | *A sudden bang in my ear* |
|  | *I know exactly what it caused and when it started* |
|  | *I had a hearing limitation on one side. My sense of direction had disappeared. The family doctor gave me nasal drips and the problem was solved.* |
|  | *I have hearing aids in both ears since 2010. My hearing was worse than my age (then 46). In 2013 the musical sounds (as I call them) started form one day. These musical sounds have expanded over the years.* |
|  | *I experience mostly at tranquil moment. after rush.* |
|  | *I think my Meniere’s disease also has an influence to this. I have not been bothered by it the last 8 to 9 months, but I think the tinnitus was caused by it.* |
|  | *The use of a hearing aid* |
|  | *Heart attack* |
|  | *I was very sick and dizzy for a whole day. The complaints increased afterwards.* |
|  | *Allergies / hay fever/ itching in the ears* |
|  |  |
|  |  |
| Auditory hallucinations (n = 6) | *Sudden bangs or other short hard sounds* |
|  | *I have also heard other sounds, like hearing one or more voices. I do not hear voices now. I learned how to cope with it.* |
|  | *Because I have single sided dearness; it is hard for me to hear where (direction) a sound is coming from* |
|  | *Hum, whiz, beep* |
|  | *Rarely, I suddenly hear a loud sirens or blaring trumpets or loud church bells. These sound have (luckily) appeared to be temporary.* |
|  | *With ambulances/flashing lights in traffic it is sometimes difficult to determine where it comes from. But I don’t think I am unique in that.* |
|  |  |
| Social position (n = 39) | *Freelancers ( n = 21)* |
|  | *Volunteers (n = 7)* |
|  | *Without income* |
|  | *Sickness law UWV* |
|  | *WWB* |
|  | *WSW through (name of company)* |
|  | *Stopped working early* |
|  | *Pre retirement* |
|  | *Part-time retired* |
|  | *Supernumerary / flexible working* |
|  | *Almost without a job (starting 22-05-2013)* |
|  | *Artist* |
|  |  |
| Please note, the answers are direct translations from Dutch. | |
